# Effects of Transcranial Direct Current Stimulation over the Left Sensorimotor Cortex on Bimanual Force Control: A Computational and Experimental Investigation

**DOI:** 10.3390/bioengineering13050502

**Published:** 2026-04-26

**Authors:** Vinicius de Moura Silva Lima, Eduarda Faria Arthur, Rafaela Rodrigues Dousseau Gonzaga, Luan Faria Diniz, Rodrigo Cunha de Mello Pedreiro, Osmar Pinto Neto

**Affiliations:** 1 Biomedical Engineering Department, Anhembi Morumbi University, São José dos Campos 12247-016, SP, Brazil; viniciuslima_15@outlook.com (V.d.M.S.L.); eduardafaria.biomed@gmail.com (E.F.A.); contatorafaeladousseau@gmail.com (R.R.D.G.); luandiniz0@gmail.com (L.F.D.); 2 Arena235Research Lab., São José dos Campos 12246-876, SP, Brazil; 3 School of Physical Education and Sports, Federal University of Rio de Janeiro, Rio de Janeiro 21941-901, RJ, Brazil; rodrigocmp1@gmail.com; 4 Physical Education Department, Estácio de Sá University, Teresópolis 25963-150, RJ, Brazil; 5 Kinesiology Department, California State University San Marcos, San Marcos, CA 92096, USA

**Keywords:** transcranial direct current stimulation, primary motor cortex, lateral premotor cortex, supplementary motor area, bimanual coordination, proprioception, force control, computational modeling

## Abstract

Transcranial direct current stimulation (tDCS) over motor–premotor regions may modulate motor performance, though underlying mechanisms remain unclear. Twenty-four athletes (9 females, 15 males) were randomly assigned to receive anodal tDCS (2 mA, 20 min) over the left sensorimotor cortex (*n* = 12) or sham stimulation (*n* = 12). Participants performed a bimanual isometric force-matching task at 30% maximal voluntary contraction, with visual feedback initially provided and then removed. Force undershoot, root mean square error (RMSE), spectral power (1–3 Hz), and inter-hand coherence were analyzed. A computational model was developed to test whether enhanced proprioceptive feedback processing could account for observed effects. Following tDCS, force undershoot decreased significantly (*p* = 0.002, *d* = −1.15) and RMSE improved (*p* = 0.010, *d* = −0.91). Spectral power in the 1–3 Hz band increased (*p* = 0.012, *d* = 0.87), suggesting enhanced corrective oscillations. These within-group changes were absent in the sham group (all *p* > 0.20), although Group × Epoch interactions did not reach significance (all *p* > 0.05), likely due to limited statistical power. Inter-hand coherence remained unchanged. The computational model demonstrated that enhanced proprioceptive feedback gain qualitatively reproduces the observed behavioral pattern. Anodal tDCS over the left sensorimotor/premotor region may enhance bimanual force control under conditions requiring proprioceptive feedback. Replication with larger samples is needed to confirm between-group specificity.

## 1. Introduction

The sensorimotor and premotor cortices, including the primary motor cortex (M1), lateral premotor cortex, and medial premotor areas, such as the supplementary motor area (SMA), are critical for motor planning, bimanual coordination, and internal representation of movement [1,2]. These regions integrate sensory information to maintain and update internal models of motor commands, particularly when external feedback is limited or absent [3]. This function is especially relevant in tasks requiring continuous force production, where proprioceptive feedback becomes essential when visual information is removed.

Transcranial direct current stimulation (tDCS) is a noninvasive neuromodulation technique that has gained considerable attention for its potential to enhance motor performance [4]. Anodal tDCS increases cortical excitability through subthreshold depolarization of neuronal membranes, leading to enhanced synaptic efficacy and potentially improved sensorimotor processing [5]. Previous research has demonstrated that tDCS over motor-related cortical areas can improve various aspects of motor function, including reaction time, motor learning, and force production accuracy [6,7].

The contribution of the premotor and medial premotor areas to proprioceptive information processing for motor control has been established through neuroimaging and lesion studies [8]. Dynamic causal modeling studies have revealed substantial input from the somatosensory areas to the premotor regions and their contribution to the integration of proprioceptive signals during force production tasks [9]. These mechanisms support the maintenance of internal representations of target forces when external feedback is unavailable, a process that relies heavily on proprioceptive recalibration [10].

Recent research from our laboratory has demonstrated the efficacy of tDCS over different nodes in the motor control network. Almeida et al., 2024 [11], showed that combining tDCS over a medial premotor montage with exercise improved mobility and tremor management in patients with Parkinson’s disease, with significant improvements in the Timed Up and Go test (mean difference = 2.498 s, *p* < 0.001) and force-tremor decoupling in the 1–4 Hz band (*p* = 0.0067). Additionally, Pedreiro et al., 2023 [12], demonstrated that anodal tDCS over the dorsolateral prefrontal cortex improved handgrip endurance in elite Brazilian Jiu-Jitsu athletes, extending the time to task failure from 64.7 ± 15.9 s to 79.6 ± 18.2 s (*p* = 0.001). These findings suggest that tDCS can enhance motor performance across different populations and task demands.

Despite the growing body of research on tDCS effects, the specific mechanisms through which stimulation of motor–premotor networks enhances sensorimotor control remain incompletely understood. Hupfeld et al. and Nomura and Kirimoto [13,14] demonstrated that anodal tDCS over the medial premotor regions improved postural stability, suggesting enhanced proprioceptive processing. However, few studies have combined experimental investigations with computational modeling to provide a mechanistic account of tDCS effects on force control.

This study aimed to investigate the effects of anodal tDCS over the left sensorimotor cortex (left central montage) on bimanual force control during a task that transitioned from vision-guided to proprioception-dependent control. We hypothesized that tDCS would improve force accuracy, specifically during the vision-off epoch, by enhancing proprioceptive feedback processing. To test the mechanistic plausibility of this hypothesis, we developed a computational model of bimanual force control that incorporates proprioceptive feedback as a key parameter. We note that tDCS effects on motor performance show considerable variability across studies [15,16], and that conventional sponge-electrode montages produce broad electric fields that stimulate distributed cortical networks rather than focal regions. We therefore frame our computational model as a qualitative, hypothesis-generating framework rather than a definitive mechanistic account and interpret our findings with appropriate caution regarding both the specificity of stimulation and the reproducibility of effects.

## 2. Materials and Methods

### 2.1. Study Design

**Figure 1:**
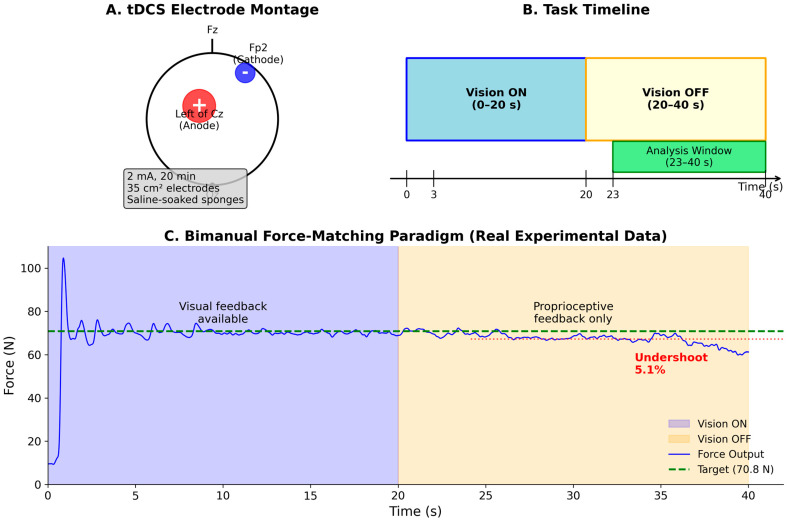
Experimental design and protocol. (**A**) tDCS electrode montage showing the anodal electrode positioned 5 cm to the left of Cz along the C-line toward C3 (left central; targeting the left sensorimotor cortex) and the cathodal electrode over Fp2 (right supraorbital region). Stimulation parameters: 2 mA intensity, 20 min duration, 35 cm^2^ electrodes with saline-soaked sponges. (**B**) Task timeline showing the Vision ON epoch (0–20 s) with continuous visual feedback and the Vision OFF epoch (20–40 s) without visual feedback. The analysis window (23–40 s, shaded green) excludes the initial 3 s transition period. (**C**) Representative force trace from a single participant showing bimanual force output relative to the target (30% MVC). During Vision OFF, force drifts below target (undershoot) when relying solely on proprioceptive feedback.

This was a randomized, sham-controlled, partially blinded, parallel-group study. Participants were randomly assigned to receive either active anodal tDCS or sham stimulation. The experimental protocol consisted of a single session including baseline assessment (PRE), 20 min of tDCS or sham stimulation, and post-stimulation assessment (POST). The primary outcomes were force undershoot and RMSE during proprioception-dependent control; secondary outcomes included spectral power (1–3 Hz) and inter-hand coherence. The experimental design and protocol are illustrated in Figure 1. Panel A shows the tDCS electrode montage, with the anode positioned over the left sensorimotor cortex (5 cm left of Cz toward C3) and the cathode over the right supraorbital region (Fp2). Panel B depicts the temporal structure of each experimental trial: participants first performed a 40 s force-matching task during the PRE assessment, then received either active tDCS or sham stimulation for 20 min, followed by an identical POST assessment. Panel C illustrates the within-trial task structure, showing the transition from Vision ON (0–20 s, with continuous visual feedback) to Vision OFF (20–40 s, proprioceptive control only). The analysis window (23–40 s) excludes the initial 3 s transition period following visual feedback removal.

### 2.2. Participants

Twenty-four healthy athletes (age: 34.9 ± 10.8 years; 9 females, 15 males) were recruited. Participants were highly active experienced practitioners (5.07 ± 1.26 training days per week, 12.93 ± 7.54 years of experience) in strength training, weightlifting, and mixed-model training [17,18], and eight participants were national-level weightlifting competitors. The exclusion criteria included a history of neurological or psychiatric disorders, contraindications to tDCS (metal implants, epilepsy, and skin lesions), and current use of medications affecting the central nervous system. All participants were right-handed, as assessed using the Edinburgh Handedness Inventory [19]. Participants were randomly assigned to either the tDCS group (*n* = 12) or the sham group (*n* = 12), and the eight national-level athletes were stratified and randomized into the study groups. The local Ethics Committee approved the study, and all participants provided written informed consent (Anhembi Morumbi University, São Paulo, Brazil; protocol code 24205419.8.0000.5492, March 2020). This study was registered in the Brazilian Clinical Trials Registry (ReBEC; registration number: RBR-2jyc55x).

#### Sample Size Determination

A priori power analysis was conducted using G*Power 3.1 [20] for repeated-measures ANOVA with within-between interaction. With an expected medium effect size (f = 0.25), α = 0.05, power (1 − β) = 0.80, two groups, four measurements (2 groups × 2 epochs), correlation among repeated measures of 0.5, and nonsphericity correction ε = 1, the analysis indicated a required total sample size of 24 participants. The achieved sample size of 24 participants (12 per group) provided an actual power of 0.82 to detect the hypothesized interaction effects.

### 2.3. Transcranial Direct Current Stimulation Protocol

tDCS was delivered using a battery-driven, constant-current stimulator. The anodal electrode (35 cm^2^) was positioned 5 cm to the left of the vertex (Cz) along the central (C) line, in the direction of C3 (left central), according to the international 10–20 EEG system [21], targeting the left sensorimotor cortex (M1/premotor region) (Figure 1). The cathodal electrode (35 cm^2^) was placed over the right supraorbital region (Fp2) of the brain. The electrodes were inserted into saline-soaked sponges. For the tDCS group, stimulation was delivered at 2 mA for 20 min with 30 s ramp-up and ramp-down periods. The electrode impedance was maintained below 10 kΩ during stimulation. For the Sham group, the stimulator was ramped up to 2 mA over 30 s and then immediately ramped down, providing the initial sensation without sustained current delivery [22].

To verify the spatial distribution of the electric field, computational modeling was performed using SimNIBS 4.5 [23] with a standard head model. The simulation was approximated by a C3-Fp2 montage that produced a broad electric field over the left sensorimotor and premotor cortices, with potential extension toward the medial premotor regions. The peak electric field magnitude over the left peri-central areas was approximately 0.15–0.20 V/m, consistent with values reported to modulate cortical excitability.

### 2.4. Experimental Task

The participants performed a bimanual isometric force-matching task using two hand-grip dynamometers (Vernier, Beaverton, OR, USA). At the beginning of each session, the maximal voluntary contraction (MVC) was determined for each hand as the highest force achieved across three maximal efforts separated by 60 s of rest. Prior to data collection, the participants completed a brief familiarization period (approximately 30 s) to become acquainted with the force-matching display.

During the experimental protocol, the participants completed one trial before (PRE) and one trial after (POST) the stimulation period. Each trial lasted 40 s and was divided into two epochs: Vision ON (0–20 s), during which participants received continuous visual feedback of their force output on a computer monitor, and Vision OFF (20–40 s), during which the visual display was occluded, and participants maintained force based solely on proprioceptive feedback. The participants were instructed to produce and maintain a combined bimanual force equal to 30% of their summed MVC. Force signals were sampled at 100 Hz using Logger Pro software (Vernier, version 3.16.2) and subsequently low-pass filtered at 15 Hz using a fourth-order Butterworth filter.

The single-trial design was chosen to minimize learning effects, as the force-matching task at 30% MVC was simple and well within the participants’ capabilities. This approach ensured that the observed changes could be attributed to the intervention rather than practice effects.

### 2.5. Outcome Measures

The analysis focused on the Vision OFF epoch, specifically the window from 23–40 s to exclude the initial transition period following visual feedback removal. All outcome measures were computed from the summed bimanual force signal (left hand + right hand).

#### 2.5.1. Force Accuracy Measures

Force Undershoot [%] = [[Target Force − Mean Force]/Target Force] × 100, representing the systematic bias below the target during proprioceptive control. Root Mean Square Error (RMSE, in Newtons] = √mean[(Force – Target)^2^], representing overall force accuracy, including both bias and variability.

#### 2.5.2. Spectral Analysis

The power spectral density was estimated using Welch’s method with the following parameters: Hanning window, segment length of 1024 samples (10.24 s at 100 Hz sampling rate), and 50% overlap between segments. This configuration yielded approximately 2–3 segments per 17 s analysis window, providing a frequency resolution of approximately 0.1 Hz. The power in the 1–3 Hz band was computed by integrating the power spectral density across this frequency range. This frequency band was selected because it corresponds to voluntary corrective adjustments mediated by sensory feedback loops [24,25].

#### 2.5.3. Inter-Hand Coherence

The magnitude-squared coherence between the left and right-hand force signals was computed using Welch’s method with matching parameters (segment length = 512 samples, 50% overlap, and Hanning window). The mean coherence was calculated for four frequency bands: 0–1 Hz (common slow drift), 1–3 Hz (corrective oscillations), 3–7 Hz (faster adjustments), and 7–12 Hz (physiological tremor range).

### 2.6. Primary and Secondary Outcomes

According to the registered protocol, the primary outcomes were force undershoot and RMSE, reflecting overall accuracy during proprioception-dependent control. Secondary outcomes included spectral power in the 1–3 Hz band (reflecting corrective oscillatory activity) and inter-hand coherence (reflecting bilateral coupling). The individual-level correlations and Vision ON vs. Vision OFF comparisons were pre-specified exploratory analyses conducted to provide a mechanistic context.

### 2.7. Statistical Analysis

The primary analysis employed a mixed-design approach with Group (tDCS vs. Sham) as the between-subjects factor and Epoch (PRE vs. POST) as the within-subjects factor. The Group × Epoch interaction was tested by comparing the PRE-to-POST change scores between groups using independent-samples *t*-tests, with the resulting t^2^ reported as F(1,22). Planned post hoc comparisons examined within-group changes using paired *t*-tests with Holm–Bonferroni correction for multiple comparisons. Effect sizes are reported as Cohen’s d computed from paired difference scores (within-group) or pooled standard deviations (between-group). As sensitivity analyses, linear mixed-effects models with participant as a random intercept were also fitted, and ANCOVA models with baseline values as covariates were used to assess the robustness of findings to pre-existing group differences. Test–retest reliability was assessed using intraclass correlation coefficients (ICC) computed from the sham group’s PRE–POST data.

To examine the mechanistic relationship between corrective oscillations and force accuracy at the individual level, Pearson correlations were computed between change scores (POST-PRE) for 1–3 Hz power and change scores for undershoot and RMSE, both across all participants and separately within each group.

To test the specificity of the tDCS effects on proprioception-dependent control, paired *t*-tests were used to compare performance during the Vision ON versus Vision OFF epochs within each Group × Epoch combination. For that, we used Vision ON from 3–20 s and Vision OFF from 23–40 s to exclude the initial and transition phases and have both conditions with 17 s.

Statistical significance was set at *p* < 0.05. All analyses were performed using Python (version 3.11) with the scipy (version 1.11), statsmodels (version 0.14), and pandas (version 2.0) libraries.

### 2.8. Computational Model

A closed-loop computational model of bimanual force control was developed to test the hypothesis that enhanced proprioceptive feedback processing underlies the observed tDCS effects. The model simulates the force production for each hand (h ∈ {L, R}) across the 40 s trial with the following mathematical formulation:

#### 2.8.1. State Equations

During Vision ON (t < 20 s), the force is controlled via visual feedback:

F_h[t + Δt] = F_h[t] + K_vis · e_h[t] + C[t] · w_c + η_motor[t] + τ_h[t](1)

where e_h[t] = T − F_h[t] is the visual error (target minus current force), K_vis = 0.12 is the visual feedback gain, C[t] is the common drive (shared bilateral input, low-pass filtered at 2 Hz), w_c = 0.15 is the common drive weight, η_motor[t] ~ N [0, σ_motor] is the motor execution noise (σ_motor = 0.22 N during Vision ON; σ_motor = 0.25 N during Vision OFF), and τ_h[t] is the physiological tremor (sinusoidal, 10–10.2 Hz).

During Vision OFF (t ≥ 20 s), the force is controlled via proprioceptive feedback:

T_int[t + Δt] = T_int[t] · [1 − λ_drift · Δt](2)

ê_h[t] = T_int[t] − F^_h[t − δ] + η_proprio(3)

F_h[t + Δt] = F_h[t] + G_proprio · K_base · ê_h[t] + C[t] · w_c + η_motor[t] + τ_h[t](4)

where T_int[t] is the internal target representation that drifts downward without visual reinforcement; λ_drift = 0.0045 · [1 − 0.5 · G_proprio] is the target drift rate (higher G_proprio = slower drift); F^_h[t − δ] is the delayed proprioceptive estimate with delay δ = 100 ms; η_proprio ~ N[0, 0.15] is the proprioceptive measurement noise; G_proprio ∈ [0.2, 0.8] is the proprioceptive feedback gain parameter; and K_base = 0.15 is the base correction gain.

#### 2.8.2. Key Model Predictions

The model predicts that increasing G_proprio (representing enhanced proprioceptive processing following tDCS) produces two concurrent effects: (1) reduced undershoot: Higher G_proprio reduces the target drift rate (λ_drift), resulting in better maintenance of the internal target representation and less systematic force undershoot. (2) Increased 1–3 Hz power: Higher G_proprio amplifies the correction term (G_proprio · K_base · ê_h), resulting in larger corrective adjustments that manifest as increased spectral power in the 1–3 Hz band.

#### 2.8.3. Between-Trial Variability

To capture realistic trial-to-trial fluctuations, the model incorporates stochastic variations in two parameters: λ_drift ~ N(λ_drift_base, 0.0008) and K_correction ~ N(G_proprio · K_base, 0.008). This variability reflects the natural fluctuations in attention, fatigue, and neural processing across trials.

#### 2.8.4. Simulation Protocol

The simulations were run at 100 Hz for 40 s, matching the experimental sampling rate and trial duration. For the dose–response analysis, G_proprio was varied from 0.20 to 0.80 in increments of 0.05–0.10, with 20 simulations per G_proprio value to characterize between-trial variability. Sham and tDCS conditions were modeled with G_proprio = 0.25 and G_proprio = 0.70, respectively, selected based on parameter search to qualitatively match experimental effect sizes.

The motor noise and proprioceptive noise parameters were selected so that the model’s Vision ON-to-OFF variability increase is consistent with the modest increase (approximately 28%) observed in the experimental RMSE data, rather than producing an unrealistically large variability jump at the vision removal transition. We note that the model serves as a qualitative, hypothesis-generating framework designed to illustrate how changes in proprioceptive feedback gain could produce the observed pattern of effects. The model’s simplified architecture, with fixed noise parameters, a single feedback delay, and deterministic common drive, does not capture the full range of inter-individual variability in force control behavior, and formal fitting of model parameters to individual participant data was not feasible. The model is therefore best interpreted as demonstrating the computational sufficiency of the proprioceptive gain hypothesis rather than providing a quantitative account of individual differences.

### 2.9. Blinding and Safety

The study employed partial blinding: participants were blinded to stimulation condition, but the experimenter administering tDCS was aware of the condition to operate the device. For the sham group, the tDCS device was ramped up to 2 mA over 30 s and then immediately ramped down, producing initial sensations (tingling, itching) comparable to active stimulation, a commonly used sham method [22]. All behavioral data were analyzed blind to group assignment in the initial analysis phase. A formal post-session blinding assessment questionnaire was not administered. Although no participant spontaneously reported awareness of their group assignment, expectancy effects cannot be definitively excluded. This is acknowledged as a limitation.

No adverse events were reported during or after the study period. All participants completed both sessions without complaints of discomfort, headache, or skin irritation at the electrode sites.

## 3. Results

The experimental results revealed a consistent pattern across primary outcomes: the tDCS group showed large, statistically significant within-group improvements in force accuracy (reduced undershoot, d = −1.15; reduced RMSE, d = −0.91) and increased corrective oscillatory activity (1–3 Hz power, d = 0.87), whereas the sham group showed no significant changes on any metric. However, Group × Epoch interactions did not reach statistical significance for any outcome, likely reflecting limited statistical power with *n* = 12 per group. Inter-hand coherence was preserved in both groups. Individual-level correlations supported the mechanistic hypothesis specifically within the tDCS group. The computational model reproduced the qualitative pattern of findings. Detailed results are presented in the following subsections.

### 3.1. Experimental Results

#### 3.1.1. Baseline Comparisons

Baseline (PRE) measures did not differ significantly between groups for undershoot (Sham: 4.40 ± 5.72%; tDCS: 4.82 ± 4.75%; t(22) = 0.21, *p* = 0.834), RMSE (Sham: 3.36 ± 2.23 N; tDCS: 3.46 ± 1.86 N; t(22) = 0.12, *p* = 0.906), or inter-hand coherence in any frequency band (all *p* > 0.20). Baseline 1–3 Hz power showed a trend toward higher values in the sham group (0.088 ± 0.102 N^2^/Hz) compared with the tDCS group (0.038 ± 0.020 N^2^/Hz; t(22) = 1.66, *p* = 0.111), although this difference did not reach statistical significance.

#### 3.1.2. Force Accuracy

The tDCS group demonstrated a significant reduction in force undershoot from PRE (4.82 ± 4.75%) to POST (0.30 ± 3.37%; paired t(11) = 3.98, *p* = 0.002, d = −1.15), while the sham group showed no significant change (PRE: 4.40 ± 5.72%; POST: 2.53 ± 5.66%; t(11) = 1.25, *p* = 0.237, d = −0.36). The Group × Epoch interaction did not reach statistical significance (F(1,22) = 2.01, *p* = 0.170), likely reflecting the high inter-individual variability in undershoot (SD = 3.4–5.7%) relative to the sample size. Similarly, RMSE decreased significantly within the tDCS group (PRE: 3.46 ± 1.86 N; POST: 2.29 ± 1.16 N; t(11) = 3.13, *p* = 0.010, d = −0.91), with no significant change in the sham group (3.36 ± 2.23 N to 2.81 ± 3.14 N; t(11) = 0.88, *p* = 0.397, d = −0.25). The RMSE Group × Epoch interaction was not significant (F(1,22) = 0.70, *p* = 0.411). Effect sizes for within-tDCS changes were large (d = −1.15 and −0.91), while within-sham changes were small (d = −0.36 and −0.25), yielding between-group effect size differences of d = 0.61 (undershoot) and d = 0.36 (RMSE).

#### 3.1.3. Spectral Power

Power in the 1–3 Hz band increased significantly within the tDCS group from PRE (0.038 ± 0.020 N^2^/Hz) to POST (0.071 ± 0.047 N^2^/Hz; t(11) = −3.01, *p* = 0.012, d = 0.87), representing an 87% increase in corrective oscillatory power. The sham group showed no significant change (PRE: 0.088 ± 0.102 N^2^/Hz; POST: 0.113 ± 0.131 N^2^/Hz; t(11) = −0.69, *p* = 0.506, d = 0.20). The Group × Epoch interaction was not significant (F(1,22) = 0.05, *p* = 0.831), and ANCOVA with baseline 1–3 Hz power as covariate confirmed that the apparent group difference in POST values was accounted for by pre-existing baseline differences (β = −0.012, *p* = 0.765). Given the limited test–retest reliability of single-trial spectral estimates (ICC = 0.43) and the baseline group imbalance, the 1–3 Hz power findings should be considered exploratory.

#### 3.1.4. Inter-Hand Coherence

**Figure 2:**
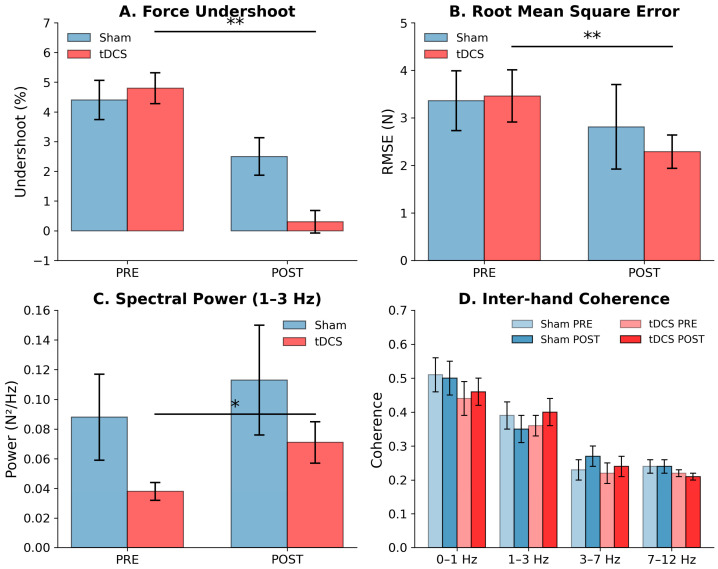
Experimental results. (**A**) Force undershoot (%) for sham and tDCS groups at PRE and POST assessments. The tDCS group showed a significant within-group reduction (paired t(11) = 3.98, * *p* = 0.002, d = −1.15); the Group × Epoch interaction was not significant (*p* = 0.170). (**B**) Root mean square error (RMSE) showing improved accuracy within the tDCS group (t(11) = 3.13, * *p* = 0.010, d = −0.91; interaction *p* = 0.411). (**C**) Spectral power in the 1–3 Hz band showing increased corrective oscillatory activity within the tDCS group (t(11) = −3.01, * *p* = 0.012, d = 0.87; interaction *p* = 0.831). (**D**) Inter-hand coherence across frequency bands showing no significant effects. Error bars represent standard error of the mean. * *p* < 0.05 and ** *p* < 0.01 for within-group paired comparisons.

No significant group × epoch interactions were observed for coherence in any frequency band (all *p* > 0.30). Coherence followed the expected physiological pattern, with the highest values in the 0–1 Hz band and no peak at the tremor frequency (7–12 Hz). The experimental results are summarized in Table 1 and Figure 2.

#### 3.1.5. Individual-Level Correlations

To examine whether changes in corrective oscillatory activity were associated with changes in force accuracy at the individual level, Pearson’s correlations were computed between change scores (POST-PRE). Across all participants, the correlation between Δ Power (1–3 Hz) and Δ Undershoot was not significant (r = 0.24, *p* = 0.268), nor was the correlation with Δ RMSE (r = 0.25, *p* = 0.230).

**Figure 3:**
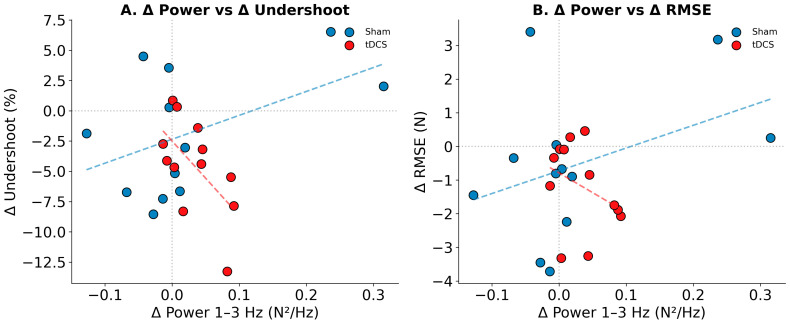
Individual-level correlations between changes in spectral power and force accuracy. (**A**) Change in 1–3 Hz power versus change in undershoot. Within the tDCS group (red solid line), increased power was significantly associated with decreased undershoot (r = −0.59, *p* = 0.042), supporting the mechanistic hypothesis. This correlation was absent in the Sham group (blue dashed line; r = 0.48, *p* = 0.115). (**B**) Change in 1–3 Hz power versus change in RMSE. Separate regression lines are shown for each group. Each point represents one participant.

However, when examined separately by group, a significant negative correlation emerged in the tDCS group between Δ Power and Δ Undershoot (r = −0.59, *p* = 0.042), indicating that participants who showed greater increases in 1–3 Hz power following tDCS also showed greater reductions in force undershoot. This association was not observed in the sham group (r = 0.48, *p* = 0.115). The opposite directions of these correlations between groups suggest that tDCS may have altered the relationship between corrective oscillations and force accuracy, consistent with the hypothesis of enhanced proprioceptive feedback processing. These correlations are illustrated in Figure 3.

#### 3.1.6. Vision ON vs. Vision OFF Specificity

To test whether the tDCS effects were specific to proprioception-dependent control, we compared performance during Vision ON (3–20 s) versus Vision OFF (23–40 s) epochs. As expected, the RMSE was consistently lower during Vision ON than Vision OFF across all conditions, reflecting the superior accuracy afforded by visual feedback. Power in the 1–3 Hz band was consistently higher during Vision ON than Vision OFF (all paired *t*-tests *p* < 0.05), likely reflecting the faster, more continuous corrections enabled by visual feedback.

Critically, the tDCS-induced improvements in RMSE were more pronounced during Vision OFF (PRE: 3.46 ± 1.86 N to POST: 2.29 ± 1.16 N; 34% improvement) than during Vision ON (PRE: 1.95 ± 1.05 N to POST: 1.90 ± 1.83 N; 3% improvement), supporting the hypothesis that tDCS specifically enhanced proprioception-dependent control.

#### 3.1.7. Sensitivity and Reliability Analyses

Test–retest reliability, assessed from the sham group’s PRE–POST intraclass correlation coefficients, was good for RMSE (ICC = 0.68), moderate for undershoot (ICC = 0.59), and fair for 1–3 Hz spectral power (ICC = 0.43). Coherence measures showed poor reliability (ICC < 0.45 across all bands), consistent with the limited spectral resolution afforded by single-trial estimates from 17 s analysis windows.

ANCOVA with baseline values as covariates was conducted for each primary metric to assess the robustness of between-group comparisons. After adjusting for baseline, the Group effect on POST values was not significant for undershoot (β = −2.45, *p* = 0.138), RMSE (β = −0.59, *p* = 0.424), or 1–3 Hz power (β = −0.012, *p* = 0.765). These results indicate that the observed within-group improvements, while consistent and large in the tDCS group, cannot be unambiguously attributed to the stimulation versus pre-existing individual differences with the present sample size.

### 3.2. Computational Model Results

The computational model successfully reproduced the qualitative pattern of the experimental results by manipulating the proprioceptive gain parameter (G_proprio). When G_proprio = 0.25 (sham condition), the model produced undershoot values of approximately 4.4%, which decreased to approximately 3.3% when G_proprio = 0.70 (tDCS condition). The between-trial variability incorporated in the model generated realistic standard deviations across the simulated trials (undershoot SD ≈ 0.9%), which was comparable to the experimental observations.

**Figure 4:**
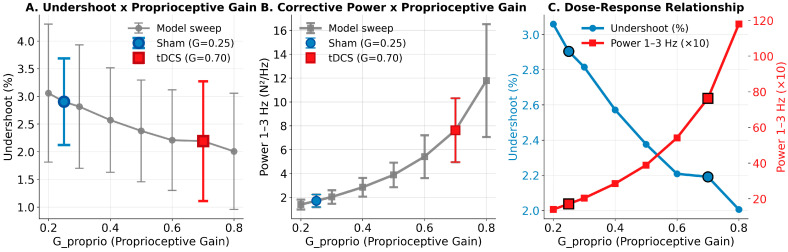
Computational model dose–response analysis. (**A**) Simulated undershoot (%) as a function of proprioceptive gain (G_proprio). Undershoot decreases monotonically with increasing G_proprio. (**B**) Simulated spectral power in the 1–3 Hz band increases with G_proprio, reflecting enhanced corrective oscillations. (**C**) Combined dose–response showing that increasing a single parameter (G_proprio) simultaneously reduces undershoot and increases corrective power, matching the experimental pattern. Large markers indicate Sham (G = 0.25, blue circle) and tDCS (G = 0.70, red square) conditions. Error bars represent standard deviation across 20 simulations per G_proprio value, reflecting modeled between-trial variability.

The model showed that increased G_proprio led to enhanced 1–3 Hz power in the force spectrum, reflecting more active corrective oscillations. Specifically, the power increased from approximately 7 (×10 N^2^/Hz) at G_proprio = 0.25 to approximately 15 (×10 N^2^/Hz) at G_proprio = 0.70. The RMSE remained relatively stable across G_proprio values, and inter-hand coherence was preserved. Dose–response analysis across G_proprio values (0.20–0.80) revealed systematic relationships: undershoot decreased monotonically with increasing G_proprio, whereas corrective power (1–3 Hz) increased. These findings demonstrate that enhanced proprioceptive feedback processing is computationally sufficient to produce the observed behavioral changes (Figure 4).

**Figure 5:**
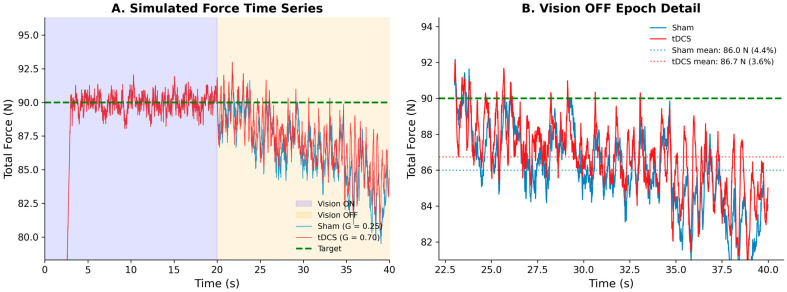
Computational model simulated time series. (**A**) Simulated total bimanual force for Sham (G_proprio = 0.25, blue) and tDCS (G_proprio = 0.70, red) conditions across the full 40 s trial, showing Vision ON (0–20 s, blue shading) and Vision OFF (20–40 s, orange shading) epochs. The green dashed line indicates the target force (90 N, corresponding to 2 × 45 N per hand). During Vision ON, visual feedback drives force to the target; upon vision removal, force drifts below the target due to internal target decay. (**B**) Detail of the Vision OFF analysis window (23–40 s). Dotted lines indicate the mean force for each condition, with the corresponding undershoot percentage. The tDCS condition (higher G_proprio) maintains force closer to the target. Note that the model captures comparable levels of force variability in both epochs, consistent with the experimental data. The model does not reproduce the full magnitude of the experimental undershoot difference (approximately 4.5% for Sham PRE vs. 0.3% for tDCS POST), as the single-parameter architecture limits the achievable separation (see Limitations).

The model successfully reproduced the qualitative pattern of experimental results by manipulating the proprioceptive gain parameter. Figure 5 shows representative simulated time series for Sham (G_proprio = 0.25) and tDCS (G_proprio = 0.70) conditions. During the Vision ON epoch, both conditions produce stable force near the target; during Vision OFF, force drifts below target, with the Sham condition showing greater undershoot (4.4% vs. 3.6% in the illustrated trial). Critically, both conditions show comparable levels of force variability across the Vision ON and Vision OFF epochs, consistent with the experimental observation that RMSE increases only modestly (approximately 28%) when visual feedback is removed.

We note that formal fitting of model parameters to individual participant data was not feasible, as the model’s simplified architecture could not reproduce the full range of observed inter-individual variability (e.g., the model’s minimum achievable RMSE exceeded the lowest observed experimental values). The model, therefore, serves as a qualitative framework demonstrating that a single-parameter change, enhanced proprioceptive feedback gain, is computationally sufficient to reproduce the direction and pattern of behavioral effects across multiple metrics simultaneously. Future work should develop more detailed, individually parameterizable models to enable formal parameter estimation and predictive validation.

## 4. Discussion

This study investigated the effects of anodal tDCS over a left-central montage (broadly targeting the left sensorimotor and premotor cortex) on bimanual force control during a task that transitioned from visual to proprioceptive feedback. The main findings were as follows: [1] the tDCS group showed large and consistent PRE-to-POST improvements in force accuracy (reduced undershoot, d = −1.15; reduced RMSE, d = −0.91) and increased 1–3 Hz spectral power (d = 0.87), whereas the sham group showed no significant changes on any metric; [2] although the Group × Epoch interactions did not reach statistical significance, likely due to limited power with *n* = 12 per group, the pattern of results was consistent across all three primary outcomes; [3] inter-hand coherence was preserved, indicating intact bilateral coupling; [4] individual-level correlations revealed a significant association between increased 1–3 Hz power and improved accuracy specifically within the tDCS group (r = −0.59, *p* = 0.042); and [5] a computational model demonstrated that enhanced proprioceptive feedback gain qualitatively reproduces the observed behavioral pattern.

### 4.1. tDCS Effects on Force Control

The improvement in force accuracy following tDCS aligns with previous research showing the beneficial effects of stimulating motor–premotor networks on motor control. Vollmann et al., 2013 [7], demonstrated that anodal tDCS over the SMA enhanced motor sequence learning, while Carlsen et al., 2015 [10], showed improvements in reaction time tasks requiring internal preparation. Our findings extend the literature by demonstrating specific improvements in proprioception-dependent force control.

The increase in 1–3 Hz spectral power following tDCS is particularly informative. This frequency band corresponds to the corrective adjustments driven by sensory feedback [24,25]. The enhanced power suggests that tDCS increased the gain of the feedback control loop, resulting in larger and more frequent corrections of the movement trajectory. Importantly, this increased corrective activity was associated with improved accuracy, suggesting functional rather than dysfunctional oscillation.

Our findings are consistent with those of a recent work from our laboratory [11], where it was observed that tDCS over the SMA combined with exercise reduced force-tremor coherence in the 1–4 Hz band in patients with Parkinson’s disease, suggesting improved decoupling between pathological tremor and intentional force production. The present results extend these findings to healthy athletes, demonstrating that tDCS enhances the functional coupling between corrective oscillations and motor output.

### 4.2. Interpretation of Statistical Findings

The pattern of the statistical results warrants careful interpretation. While the Group × Epoch interactions did not reach conventional significance thresholds for any individual metric (undershoot: F(1,22) = 2.01, *p* = 0.170; RMSE: F(1,22) = 0.70, *p* = 0.411; 1–3 Hz power: F(1,22) = 0.05, *p* = 0.831), the convergent pattern across all three primary outcomes is notable: the tDCS group consistently showed large within-group effects (d = 0.87–1.15) that were absent in the sham group (d = 0.20–0.36). The a priori power analysis indicated 82% power to detect a medium interaction effect (f = 0.25); however, the observed high inter-individual variability in undershoot (SD = 3.4–5.7%) and the modest sample size (*n* = 12 per group) likely contributed to insufficient power for detecting significant interactions. Sensitivity analyses using ANCOVA with baseline covariates further showed that between-group comparisons were not robust to adjustment for pre-existing differences, particularly for 1–3 Hz spectral power where a baseline imbalance was present.

Importantly, the significant within-group correlation in the tDCS group (r = −0.59, *p* = 0.042) between Δ Power and Δ Undershoot provides individual-level evidence supporting the mechanistic hypothesis, independent of the between-group comparisons. This correlation was absent in the sham group, suggesting a tDCS-specific alteration in the relationship between proprioceptive feedback processing and motor accuracy.

### 4.3. Mechanistic Interpretation

The computational model provides a qualitative mechanistic framework for understanding how tDCS may affect proprioceptive force control. By manipulating a single parameter (proprioceptive gain, G_proprio), the model reproduces the qualitative direction of the experimental findings: reduced undershoot and increased 1–3 Hz corrective oscillations (Figure 4), with simulated time series showing comparable force variability across visual and proprioceptive control epochs (Figure 5). This parsimony supports the hypothesis that tDCS over the left sensorimotor/premotor region may enhance proprioceptive processing. However, we emphasize that the model parameters were selected to qualitatively match observed effects and were not formally fitted to individual participant data, as the model’s simplified architecture could not capture the full range of inter-individual variability. Alternative mechanistic hypotheses—including altered common neural drive, changes in attention or arousal via prefrontal engagement from the broad montage, or modified internal model updating—cannot be excluded based on the present data.

The inclusion of between-trial variability in the model was essential for generating realistic standard deviations that matched the experimental observations. This variability, implemented through stochastic fluctuations in the drift rate and correction gain, reflects the natural trial-to-trial variations in factors such as attention, fatigue, and neural noise. The model’s ability to capture both the mean effects and variability strengthens the mechanistic interpretation.

The proposed mechanism is consistent with the known SMA functions. The SMA receives proprioceptive input and contributes to state estimation during motor control [2,26]. By increasing cortical excitability, anodal tDCS may enhance proprioceptive signal processing, leading to more accurate state estimates and better maintenance of internal target representations [13,14].

Individual-level correlation analyses provide additional support for this mechanistic hypothesis. Within the tDCS group, participants who showed greater increases in 1–3 Hz power also showed greater reductions in undershoot (r = −0.59, *p* = 0.042), suggesting a functional link between enhanced corrective oscillations and improved accuracy. Notably, this correlation was absent in the sham group (r = 0.48, *p* = 0.115, opposite direction), indicating that tDCS may have fundamentally altered the relationship between proprioceptive feedback processing and motor output.

We note that the baseline imbalance in 1–3 Hz spectral power between groups (sham: 0.088 ± 0.102 N^2^/Hz; tDCS: 0.038 ± 0.020 N^2^/Hz) limits mechanistic inference from spectral changes. ANCOVA controlling for baseline confirmed that POST differences were not significant after adjustment (β = −0.012, *p* = 0.765), and spectral findings should therefore be considered exploratory. It is also possible that the observed effects involve weighted summation of multiple neural drives during movement programming, as recently described in the literature [27]. In this framework, tDCS may modulate not only proprioceptive feedback gain but also the relative weighting of visual, vestibular, and motor efference copy signals that converge on premotor planning regions. Our computational model, while focused on proprioceptive gain for parsimony, does not exclude such multi-drive integration mechanisms. Future studies employing multi-modal sensory perturbations could help dissociate these contributing factors.

The preservation of inter-hand coherence following tDCS indicates that the bilateral coordination mechanisms remain intact. This is important because it suggests that tDCS enhances individual hand control without disrupting the shared neural commands that coordinate bimanual actions. The coherence spectrum showed highest values at low frequencies (0–1 Hz), reflecting common slow drift, and no peak at tremor frequencies (7–12 Hz), consistent with independent peripheral tremor generation.

### 4.4. Stimulated Area

The selection of the left sensorimotor and premotor cortex as the stimulation target is consistent with the established role of these regions in motor planning, bimanual coordination, and internal representation of movement, particularly in tasks that rely on proprioceptive feedback when visual feedback is reduced or absent. The primary motor cortex (M1) controls voluntary movements and exhibits a distributed functional organization and experience-dependent plasticity, as evidenced by changes in cortical representations associated with practice and everyday motor use [28].

In addition, medial premotor regions, including the supplementary motor area (SMA) and pre-supplementary motor area (pre-SMA), are anatomically located near the cingulate gyrus and maintain connections with motor and subcortical regions involved in voluntary action control [2]. Studies on non-invasive brain stimulation indicate that transcranial direct current stimulation (tDCS) can induce changes in cortical excitability through subthreshold polarization of neuronal membranes, thereby modulating neural activity and synaptic efficacy [29]. In the sports context, stimulation of the motor cortex has been associated with improvements in motor performance accompanied by increases in supraspinal and spinal excitability in trained athletes [30].

### 4.5. Comparison with Previous tDCS Studies in Athletes

The present findings complement those of previous research on tDCS effects in athletic populations. de Pedreiro et al. (2023) demonstrated that anodal tDCS over the left dorsolateral prefrontal cortex improved handgrip endurance in elite Brazilian Jiu-Jitsu athletes without affecting maximal voluntary contraction [12]. Similarly, our results showed tDCS-induced improvements in the accuracy of sustained force control without changes in the baseline force production capacity. These converging findings suggest that tDCS may primarily affect the feedback control and endurance aspects of motor performance rather than the maximal force generation.

**Figure 6:**
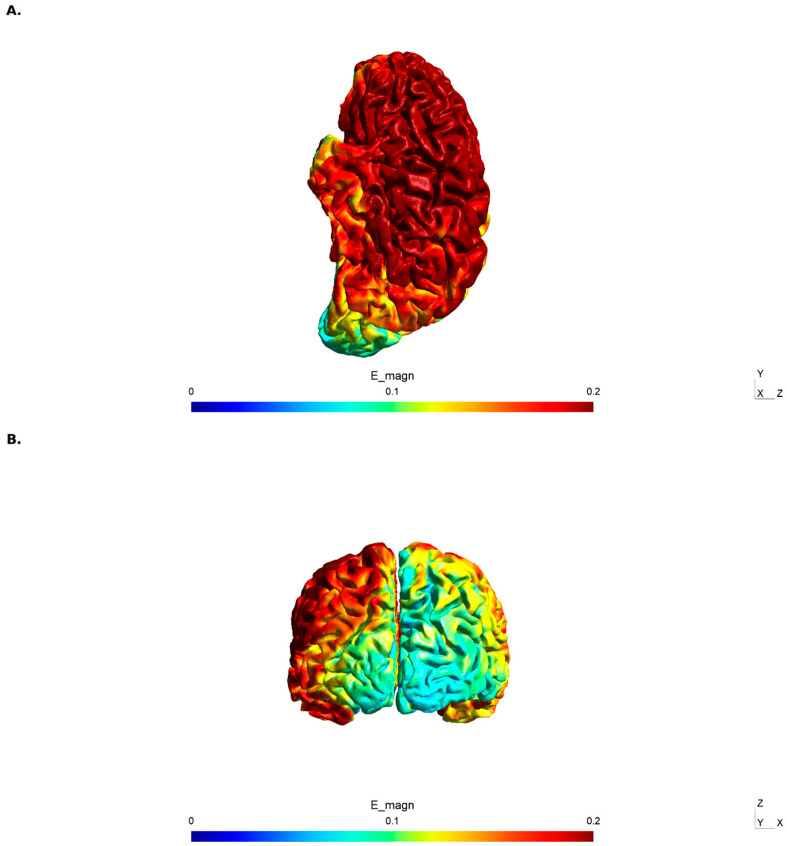
SimNIBS electric field modeling of the tDCS montage. (**A**) Left lateral view showing the distribution of electric field magnitude [|E|] across the cortical surface. Peak field intensities (0.15–0.20 V/m) are observed over the left frontal and peri-central cortex, spanning the precentral and postcentral gyri beneath the anode position. The field decreases toward the occipital and inferior temporal regions. (**B**) Posterior view confirming the lateralized field distribution, with substantially stronger magnitudes over the left hemisphere compared with the right. The broad electrode montage (anode: 35 cm^2^ sponge, 5 cm left of Cz toward C3; cathode: 35 cm^2^ sponge over Fp2) produces a distributed electric field spanning the left primary motor cortex, premotor cortex, and adjacent regions, rather than focal stimulation of any single cortical area. Color scale represents electric field magnitude in V/m (range: 0–0.2 V/m). Modeling was performed using SimNIBS 4.5 with a standard head model [Ernie] and 2 mA stimulation intensity.

The choice of a left-central montage in the present study was guided by the central role of the left sensorimotor and premotor cortices in bimanual force production and online error correction [1]. These regions contribute to maintaining internal representations of target forces and integrating proprioceptive feedback when visual information is unavailable [8,9,10]. Because conventional sponge tDCS produces a broad electric field, we complemented the experimental protocol with finite element modeling to characterize the expected spatial distribution of stimulation (Figure 6).

### 4.6. Limitations and Future Directions

This study has several limitations that should be considered when interpreting the findings. First, the sample size (*n* = 12 per group) was sufficient to detect large within-group effects but provided limited statistical power for Group × Epoch interaction tests. The non-significant interactions, despite consistent large within-tDCS effect sizes (d = 0.87–1.15), likely reflect this power limitation combined with high inter-individual variability in proprioceptive force control (undershoot SD = 3.4–5.7%). Future studies should employ larger samples (e.g., *n* = 25–30 per group) to provide adequate power for interaction effects.

Second, participants were blinded to stimulation condition, and initial data analysis was conducted blind to group assignment; however, formal post-session blinding assessment questionnaires were not administered. Although the sham protocol [30 s ramp-up/ramp-down] is designed to be perceptually indistinguishable from active stimulation, and no participant spontaneously reported awareness of their condition, expectancy effects cannot be definitively excluded. Future studies should include formal blinding assessments and report their outcomes. Furthermore, the non-blinded experimenter during stimulation delivery introduces potential for experimenter expectancy bias, which cannot be definitively excluded despite behavioral data being analyzed blind to group assignment in the initial analysis phase.

Third, with only one trial per epoch, trial-to-trial variability within individuals could not be assessed. Test–retest reliability was moderate for the primary behavioral outcomes (undershoot ICC = 0.59; RMSE ICC = 0.68) but only fair for spectral measures (1–3 Hz power ICC = 0.43) and poor for coherence (ICC < 0.45). These reliability estimates, based on single 17 s analysis windows with 2–3 spectral segments, suggest that the spectral and coherence findings should be interpreted with caution. Future studies should consider multiple trials to improve measurement precision.

Fourth, the tDCS montage (anode positioned left of Cz toward C3, cathode at Fp2, 35 cm^2^ sponge electrodes) stimulates a broad sensorimotor–premotor–prefrontal network, as confirmed by our SimNIBS modeling on a template head. Claims regarding stimulation of specific cortical areas (e.g., SMA, primary motor cortex) cannot be made with confidence. Future studies using high-definition tDCS (e.g., 4 × 1 HD-tDCS) with individualized finite element modeling could improve spatial specificity and help adjudicate between contributions from M1, lateral premotor cortex, SMA, and prefrontal regions.

Fifth, ANCOVA sensitivity analyses revealed that between-group comparisons were not robust to adjustment for baseline values, particularly for 1–3 Hz spectral power, where a pre-existing baseline imbalance was present. While this does not invalidate the within-group findings, it underscores the need for replication with balanced baseline characteristics or larger samples that can accommodate covariate adjustment.

Sixth, the computational model serves as a qualitative framework and was not formally fitted to individual data. The model’s simplified architecture could not reproduce the full range of observed behavioral variability, precluding individual-level parameter estimation. Future work should develop more detailed models incorporating individual-specific motor noise, sensory weighting, and attentional dynamics to enable formal parameter estimation, Bayesian fitting, and predictive validation.

Seventh, the sample was limited to trained athletes, and potential moderating factors such as sex (9 females, 15 males), training status, or baseline proprioceptive acuity were not explored. These factors may influence both proprioceptive processing and responsiveness to neuromodulation.

Finally, the variability and reproducibility challenges that characterize the broader tDCS literature [15,16] warrant caution in interpreting the present findings. While the consistent pattern of large within-group effects across multiple metrics is encouraging, replication with larger samples, pre-registered primary outcomes, multiple trials, and formal blinding assessment is necessary to establish the robustness of these effects.

## 5. Conclusions

Anodal tDCS delivered with a left-central montage produced large and consistent within-group improvements in bimanual force control under conditions requiring proprioceptive feedback, including reduced force undershoot (d = −1.15), improved accuracy (d = −0.91), and enhanced corrective oscillations (d = 0.87). Although Group × Epoch interactions did not reach statistical significance with the present sample size, the convergent pattern of effects across multiple metrics, absent in the sham group, provides preliminary evidence for a tDCS-specific effect. A computational model demonstrated that enhanced proprioceptive feedback gain qualitatively accounts for the observed behavioral pattern. This combined experimental and computational approach offers a hypothesis-generating framework for understanding how tDCS over the sensorimotor/premotor cortex may enhance proprioceptive control, with potential implications for rehabilitation and performance contexts. Replication with larger samples, pre-registered outcomes, and formal blinding assessment is recommended.

## Figures and Tables

**Table 1:** Summary of statistical results for primary outcome measures.

Metric	Sham PRE	Sham POST	tDCS PRE	tDCS POST	tDCS *d*	Interact. *p*	tDCS *p*
Undershoot (%)	4.40 ± 5.72	2.53 ± 5.66	4.82 ± 4.75	0.30 ± 3.37	−1.15	0.170	0.002 *
RMSE (N)	3.36 ± 2.23	2.81 ± 3.14	3.46 ± 1.86	2.29 ± 1.16	−0.91	0.411	0.010 *
Power 1–3 Hz	0.088 ± 0.102	0.113 ± 0.131	0.038 ± 0.020	0.071 ± 0.047	0.87	0.831	0.012 *
Coh 0–1 Hz	0.51 ± 0.17	0.50 ± 0.17	0.44 ± 0.17	0.46 ± 0.14	0.09	>0.30	ns
Coh 1–3 Hz	0.39 ± 0.13	0.35 ± 0.13	0.36 ± 0.09	0.40 ± 0.14	0.21	>0.30	ns

Note: Values are mean ± SD. tDCS *d* = within-group Cohen’s d (POST vs. PRE). Interact. *p* = Group × Epoch interaction. tDCS *p* = within-tDCS paired *t*-test, Holm-corrected. * *p* < 0.05; ns = non-significant.

## Data Availability

Anonymized experimental data and computational model code are available at Zenodo (DOI: 10.5281/zenodo.19745973) and can also be obtained upon request from the corresponding author.

## Abbreviations

The following abbreviations are used in this manuscript:

ANCOVA	Analysis of Covariance
C3	Central 3 (EEG electrode position)
Cz	Central Zero/Vertex (EEG electrode position)
DLPFC	Dorsolateral Prefrontal Cortex
EEG	Electroencephalography
Fp2	Frontopolar 2 (EEG electrode position)
HD-tDCS	High-Definition transcranial Direct Current Stimulation
ICC	Intraclass Correlation Coefficient
M1	Primary Motor Cortex
MVC	Maximal Voluntary Contraction
pre-SMA	Pre-Supplementary Motor Area
ReBEC	Brazilian Registry of Clinical Trials (Registro Brasileiro de Ensaios Clínicos)
RMSE	Root Mean Square Error
SMA	Supplementary Motor Area
tDCS	Transcranial Direct Current Stimulation
